# NO-cGMP signalling and cancer therapy

**DOI:** 10.1186/2050-6511-14-S1-P9

**Published:** 2013-08-29

**Authors:** Ka Bian, Alex Sotolongo, Huy Lam, Haijie Xiao, Dandan Zhang, Jun Liu, Ferid Murad

**Affiliations:** 1 Department of Biochemistry and Molecular Medicine, George Washington University,2300 I Street, NW; Ross Hall 543; Washington, DC 20037, USA

## Background

The nitric oxide (NO) and 3’,5’-cyclic guanosine monophosphate (cGMP) pathway is one of the best characterized signalling cascades that plays a central role in several physiological processes such as vasodilation, neurotransmission, and embryonic development. Soluble guanylyl cyclase (sGC) is the key receptor for NO with α1β1 as predominate heterodimer for the function. Nitric oxide binds to the ferrous heme at histidine 105 of the β1 subunit and leads to at least a 200-fold increase in sGC activity and cGMP production [1]. On the other hand, the effects of NO can be attributed to cGMP-independent pathway which is mainly mediated by reactive oxygen/nitrogen species such as highly reactive peroxynitrite (ONOO-) [2]. The role of NO and cGMP signalling in tumour biology has been extensively studied during the past three decades, however a consensus regarding the precise role that the NO/cGMP signalling axis plays in neoplastic transformation has not been reached. Simple applications of NO or cGMP regulating reagents to various cancer cell lines or animal models has generated controversial results. We suggest several factors are contributing to this ambiguity: First, although the NO participates in normal signalling (e.g., vasodilatation and neurotransmission), NO is also a cytotoxic or apoptotic molecule when produced at high concentrations by inducible nitric oxide synthase (iNOS or NOS-2). Also, the cGMP-dependent (NO/sGC/cGMP pathway) and cGMP-independent (NO/oxidative pathway) components may vary among different tissues and cell types. Furthermore, solid tumours contain two compartments: the parenchyma (neoplastic cells) and the stroma (nonmalignant supporting tissues including connective tissue, blood vessels, and, inflammatory cells) with differing NO biology. Therefore, the NO/sGC/cGMP signalling molecules in tumours as well as the surrounding tissue must be further characterized before targeting this signalling pathway for tumour therapy.

## Results

**Figure 1:**
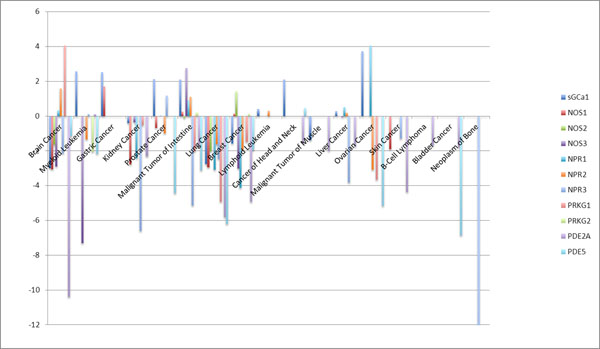
The changes of NO-cGMP signalling molecules in different human cancers compared to correspond normal tissues**.**

Our database analysis shows that by comparing with respective normal tissues, the expression of NO-cGMP signalling molecules in different human cancers varies significantly (Figure 1). The expression of NO synthase (NOS)-1 is down-regulated in brain, kidney and lung cancer specimens. NOS-2 is up-regulated in breast cancer but decreased in lung cancer. NOS-3 is higher in malignant intestine tumour, but inhibited in both brain and breast cancers. Despite the fact that sGC is an obligate heterodimer for function, sGC subunits are differentially expressed in cancers with sGCα1 being lower in brain, lung and breast cancers, but higher in prostate and ovarian cancers while sGCβ1 is attenuated in brain, lung, liver, breast cancers, but elevated in lymphoma, ovarian, head and neck cancers. The data regarding membrane bound guanylyl cyclases shows that NPR2 is up-regulated in brain cancer with non-change of NPR1; NPR1 is higher and NPR2 is lower in ovarian cancer; both NPR1 and NPR2 are down-regulated in breast cancer; and NPR1 expression is diminished in lung and kidney cancers. As the key molecules downstream of cGMP, PRKG1 is over-expressed in brain cancer and decreased in lung, breast and ovarian cancers. PRKG2 is only changed in myeloid leukaemia. As the major cGMP specific phosphodiesterase, PDE-5 shows broad depression in brain, lung, prostate, breast, malignant tumour of Intestine and myeloid leukaemia.

We have performed initial experiments to prove our hypothesis that restoration of normal NO-cGMP signalling blocks the aggressive course of cancer [3]. Pharmacologically manipulating endogenous cGMP generation in glioma cells through either stimulating pGC (NPR1 and NPR2) by ANP/BNP, or blocking PDE-5 by IBMX/ zaprinast caused significant inhibition of proliferation and colony formation of glioma U87 cells. Genetically restoring sGC expression also inversely correlated with glioma cells growth. Orthotopic implantation of glioma cells transfected with an active mutant form of sGC (sGCα1β1cys-105) in athymic mice increased the survival time by 4-fold over the control. Histological analysis of xenografts overexpressing α1β1cys-105 sGC revealed changes in cellular architecture which resemble the morphology of normal cells. In addition, a decrease in angiogenesis contributed to glioma inhibition by sGC/cGMP therapy.

## Conclusion

Our study proposes a new concept that suppressed expression of sGC a key enzyme in the NO/cGMP pathway, may be associated with an aggressive course of glioma. The sGC/cGMP signalling-targeted therapy may be a favourable alternative to chemotherapy and radiotherapy for glioma and perhaps other tumours such as breast, lung and pancreatic cancers.
